# Veterinary Public Health Activities and Management of the Livestock Sector during Earthquakes and Snowstorms in the Abruzzo Region—Italy, January 2017

**DOI:** 10.3390/ani8110218

**Published:** 2018-11-21

**Authors:** Paolo Migliaccio, Maria Nardoia, Luigi Possenti, Paolo Dalla Villa

**Affiliations:** Istituto Zooprofilattico Sperimentale dell’Abruzzo e del Molise “G. Caporale”, Campo Boario, 64100 Teramo, Italy; l.possenti@izs.it (L.P.); p.dallavilla@izs.it (P.D.V.)

**Keywords:** veterinary services, animal welfare, non-epidemic emergencies, disaster management, earthquake

## Abstract

**Simple Summary:**

This article describes the actions that were taken to provide assistance to farm animals during and in the aftermath of earthquakes and snowstorms that hit the Abruzzo region in January 2017. This experience showed the importance of an integrated emergency management response system. An updated information system, in addition to punctual data management, allows for the rationalization of resources and the organization of necessary interventions. This knowledge will be fundamental to the involvement and integration of Regional Veterinary Services (RVS) and the relevant actors in animal welfare in emergency management plans.

**Abstract:**

In 2017 the Abruzzo region, located in central Italy, was struck by a sequence of four moderately powerful seismic events (5.0 magnitude on Richter scale), in addition to heavy snowfall that caused a state of emergency affecting the resident population and farm animals. A total of 282 stables were reported to have collapsed or been damaged and several animals (224 cows, 4025 ewes, 40,725 chickens, 22 horses and 3092 pigs) were killed. The Istituto Zooprofilattico of Abruzzo and Molise “G. Caporale” (IZSAM)—National Reference Center for Veterinary Urban Hygiene and Non-Epidemic Emergencies (IUVENE), played a crucial role in planning, coordinating and controlling veterinary activities during these catastrophic events. Operational and information tools were used to manage the needs of the communities involved, as well as to prioritize the veterinary interventions, record information, provide real-time data access, and produce reporting maps and Geographic Information System (GIS) layers. These events have highlighted how the integration of veterinary services into disaster management efforts can play an important role in protecting the health and welfare of animals, whilst also restoring economic activity and community life.

## 1. Introduction

Disasters of all types, including natural ones, often jeopardize the economy, food security, and the health of humans and animals (Sen and Chander, 2003; Vroegindewey, 2014). Such events may cause significant damages to goods and the severe loss of human and animal life, both for companion animals and livestock. Nearly three million chickens and turkeys, more than thirty thousand pigs and other farmed animals died during Hurricane Floyd in North Carolina in 1999 [1]. In 1999, more than one thousand horses were killed in the wake of Hurricane Andrew in Florida and almost thirty-five thousand dairy cows froze to death in 2016 during a blizzard in Texas [2]. More recently in 2017, thousands of chickens and pigs drowned in floods after Hurricane Matthew hit North Carolina [3].

Despite the importance of the livestock sector’s contribution in raising productivity and providing a continuous stream of food and income for homes [4], farmed animals are often abandoned or left vulnerable to the dangers posed by natural disasters, with major risks for their health and welfare [5]. This is because attention usually focuses primarily on saving human life and, in many cases, because of the size of the animals and the requirements needed to transport and shelter them is prohibitive.

During and immediately after natural disasters farm animals often experience a lack of feed and water, along with severe repercussions in terms of reduced production and performances. Moreover, diseases may spread affecting livestock during and after a disaster [6]. Unlike normal climatological conditions, the scarcity of water forces domestic and wild animals to drink from the same source, which can be potentially contaminated or infected. These conditions foster the spread of diseases favoring the dissemination of infectious agents from wild to domestic biological cycles. If zoonotic agents are involved, public health could be at risk. Moreover, due to the role played by animals in affected communities [7], non-epidemic emergencies can negatively affect the wellbeing of every individual and their ability to make a contribution to said community [6].

Over time, natural disasters have highlighted the need to include livestock in contingency plans and enhance the integration of Veterinary Services by adopting emergency management and risk reduction plans at the national level. Despite international organizations, such as the World Organization for Animal Health (OIE), that have developed specific recommendations for animals in disasters [8], much work is still needed to alleviate the suffering experienced by many animals during and following a natural disaster, and to ensure their full integration into disaster preparedness and emergency management activities.

## 2. The Abruzzo Snowstorms and Earthquake

On January 2017 the Abruzzo region, the province of Teramo in particular, was affected by a sequence of natural disasters (tremors, freezing temperatures, snowstorms and avalanches). Exceptionally intense snowfall (over 2.5 m), with very low temperatures and high winds were reported.

Entire rural communities remained isolated for several days, and power and phone services were disrupted. Consequently, power supplies were disabled due to problems in the high voltage networks and in the “primary cabins” that remained out of service.

Moreover, rescue efforts were hampered due to difficulties clearing roads, with larger and heavier vehicles being unable to get through.

**Figure 1:**
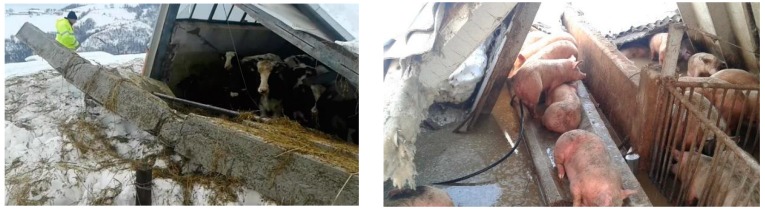

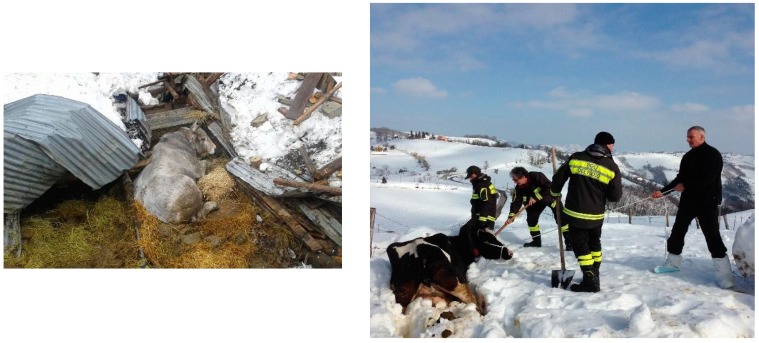
Collapsed sheds in damaged farms in the Province of Teramo and farm animals rescue. Source: Istituto Zooprofilattico Sperimentale dell’Abruzzo e del Molise “G. Caporale”.

On the morning of 18 January, four strong earthquakes occurred at close range with magnitudes ranging from 5 to 5.3 on the Richter scale and with epicenters in the province of L’Aquila (AQ), further aggravated the situation. The seismic sequence hit a series of major hills throughout the area and severely affected the animal production sector. The seismic shocks led to the structural collapse of sheds already weighed down by snow, especially in those farms located above 600 m above sea level. (Figure 1).

Thousands of livestock animals were trapped under collapsed buildings, injured or killed. The number of livestock farms that reported collapsed or damaged stables in the four provinces of Abruzzo was 282. Most of them (87%) were located in the province of Teramo (Table 1).

Moreover, on the basis of the reports registered in the IUVENE information system by the Local Health Unit (LHU) official veterinarians, it can be determined that the following number of animals were lost: 224 cows of which 216 in the province of Teramo and 8 in the province of L’Aquila; 4025 ewes: 736 of which in the province of Teramo, 3052 in the province of Pescara and 237 in the province of L’Aquila; 40,725 chickens, 22 horses, 3092 pigs all in the province of Teramo (over 13,500 pigs were present in the farms inspected in the province of Teramo).

For several days the lack of electricity caused serious difficulties to farms and processing companies, which had to use electric generators to heat water and feed the milking systems in order to maintain the continuity of production.

## 3. Measures Taken to Manage the Emergency

### 3.1. Emergency Management Organisation

The Italian “civil protection system” relies on different governmental services and agencies, including the National Health Service, in which veterinary services operate at central, regional and local levels. Within this system, the Civil Protection Department has a guiding role, in agreement with regional and local governments, of projects and activities for the prevention, forecast and monitoring of risks and intervention procedures that are common to the whole mechanism.

In the event of natural disasters, the organization of the response is normally articulated into 14 different sectors or “functions”, each with their own specific specialization, involved in the different stages of the emergency management cycle (mitigation—preparedness—response—recovery), and a series of operational structures (Table 2).

Function 2—“Health, social and veterinary assistance” is meant to coordinate and organize veterinary interventions in the field of animal welfare, animal health, food and feed safety, and veterinary public health. These activities are primarily guaranteed by the structures of the National Health Service, including central, regional and Local Veterinary Services (LVS).

At a local level, the activities of forecasting, prevention and the mitigation of risks are mayoral responsibilities. At a provincial level, the emergency management activities are under the jurisdiction of prefects, who can activate the Provincial Coordination Assistance Center (CCS), an organizational and operational center able to harmonize the actions of the local components of the civil protection operational structures.

On 17 January 2017 the prefect of Teramo, the most affected province in the Abruzzo region, ordered the activation of the CCS. Once the severity of the events had been established, IUVENE was convened on 19 January 2017, together with representatives of the LHU of Teramo and regional agricultural organizations, in order to take stock of the complexity of the situation.

Serious difficulties were identified for the agricultural sector, in particular concerning the livestock sector in the most isolated fractions, located in the most severely affected areas of the largely mountainous territory.

**Figure 2:**
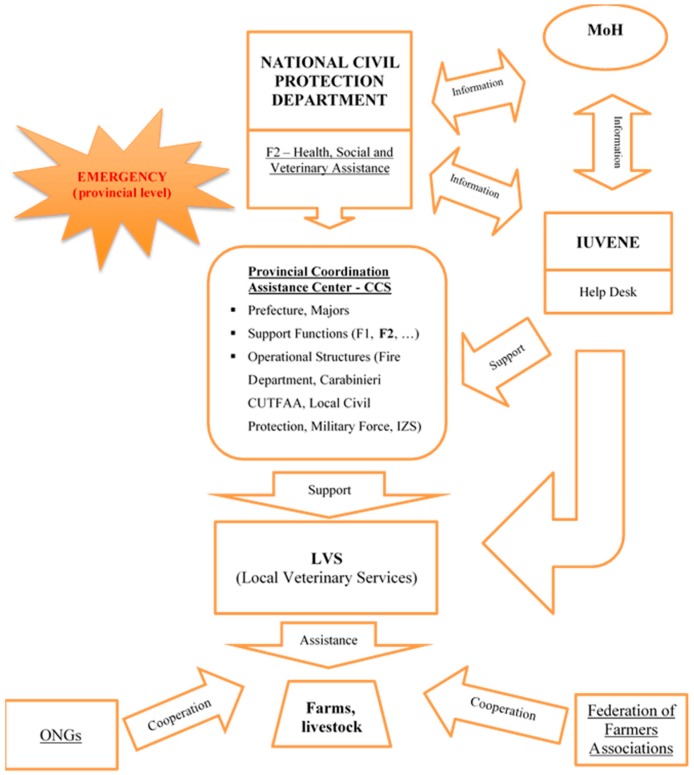
A flow chart outlining the reporting structure for animal health services in Italy.

On 21 January 2017, on the initiative of the Abruzzo region the “coordination table for zootechnical and veterinary emergencies” was established in support of the CCS and the emergency activities were carried out by the LVS under the coordination of the Comando Unità per la Tutela Forestale, Ambientale e Agroalimentare of Carabinieri (CUTFAA) of Teramo, in collaboration with the LHU of Teramo and thanks to the technical and operational support of the IUVENE staff (Figure 2).

### 3.2. Helpdesk Organisation and Field Activities

From 19 January 2017 the prefect of Teramo decreed the immediate extension of working hours from 8:00 a.m. to 8:00 p.m., including during public holidays, of the help desk connected to the IUVENE information system, to which citizens and breeders could turn, including for reports of lost and found animals.

From the outset, over 60% of the calls concerned reports of collapsed stables, requests for technical and logistic support, veterinary assistance, the rescue of injured animals, and the removal and destruction of dead animals. Requests for zootechnical feed supply as a result of collapsed or unreachable sheds or interruption of communication routes were also received.

Inspections were immediately organized in those farms that reported problems, and priority was given to injured and trapped animals.

Farms were inspected by the Local Veterinary Services of the LHU of Teramo, in collaboration with support teams provided by the Istituto Zooprofilattico of Abruzzo and Molise (IZSAM) and the University of Teramo—Faculty of Veterinary Medicine, on the basis of specific checklists developed for the assessment of the damages.

In many cases the difficulty of providing assistance to animals due to problems in the communication routes and collapsed stables was overcome thanks to the logistical support of the Fire Department and Local Civil Protection of Teramo.

A total of 633 requests came to the IUVENE help desk, and they were categorized by type, degree and urgency (need for health interventions, reports of the collapse of stables and sheds, presence of dead or injured animals, dead animal removal requests) in order to verify the critical issues highlighted by the farmers and to evaluate the outcome and the progress of the activities carried out (Table 3).

The collection and management of reports received by the IUVENE help desk enabled the coordination and execution of the following activities:

- Identification of needs, critical issues, prioritization of rescue activities in coordination with the other operational structures of the CCS (Fire Department., Italian Armed Forces, National Corps for Mountain and Speleological Rescue);
- Transfer of farm animals from collapsed sheds to temporary stables made available by other farmers;
- Preventive inspections aimed at verifying the suitability of temporary stables;
- Land mapping of livestock farms based on coordinates from National Data Bank (BDN) and altitude;
- Transmission of daily activity report to the Prefect of Teramo;
- Coordination meetings held in the presence of other operational functions in the CCS.

### 3.3. Assistance Activities to Zootechnical Farms

In Italy, emergency rescue and veterinary assistance activities are financed by state law and carried out from the National Civil Protection Department through the national health system. However, due to the severity of the situation, additional help was needed on this occasion from individual citizens, voluntary and category associations, who donated animal feed and shelter to affected livestock. Furthermore, IUVENE provided logistical and operational support in the distribution of farm feed and forage to the farmers reporting urgent supply problems (collapsed sheds, and inaccessibility of barns). All help offers received via the help desk were managed by the Comando Unità per la Tutela Forestale, Ambientale e Agroalimentare of Carabinieri (CUTFAA) and were transmitted to the CCS established in the other three provinces of Abruzzo.

**Figure 3:**
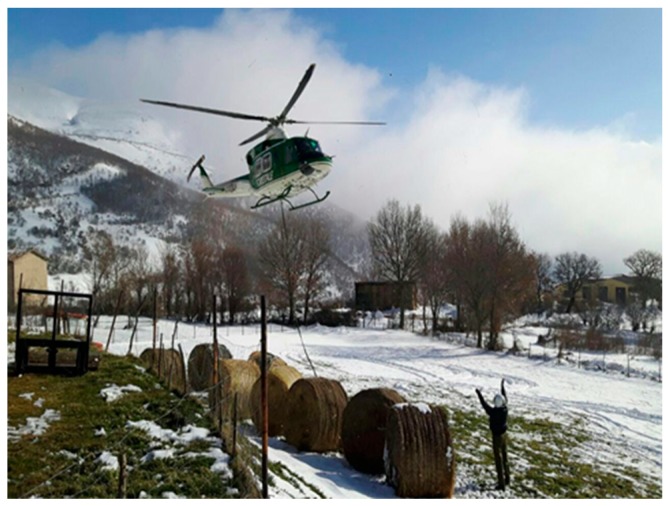
Distribution of farm feed to farms reporting urgent supply needs. Source: Istituto Zooprofilattico Sperimentale dell’Abruzzo e del Molise “G. Caporale”.

The requests of assistance for farm feed, forage and hay supply received by the help desk allowed a total of 42,483 farm animals to be assisted, of which 9291 were located in the province of L’Aquila, 664 in Chieti, 317 in Pescara and 32,211 in Teramo. The latter of these included 5079 cattle, 18,536 sheep and goats, 6820 pigs, 696 horses and 18,536 chickens. All distributions were carried out under the coordination of the Carabinieri of CUTFAA and in collaboration with the Civil Protection of Teramo—CIVES and the Agriculture Department, by creating a storage center at the CIVES headquarters. Two other storage and distribution centers, activated by the Regional Federation of Farmers, remained active until 6 February. Up until 16 February 2017, 445 deliveries of farm feed were provided by national and international donors, for a total of 4079 tons of hay, 1048 tons of grain and feed and 1114 tons of straw. The distribution was also made by air using helicopters made available by Carabinieri of CUTFAA, Italian Military Forces and Guardia di Finanza or, whenever feasible, by land trough Civil Protection vehicles (Figure 3).

**Figure 4:**
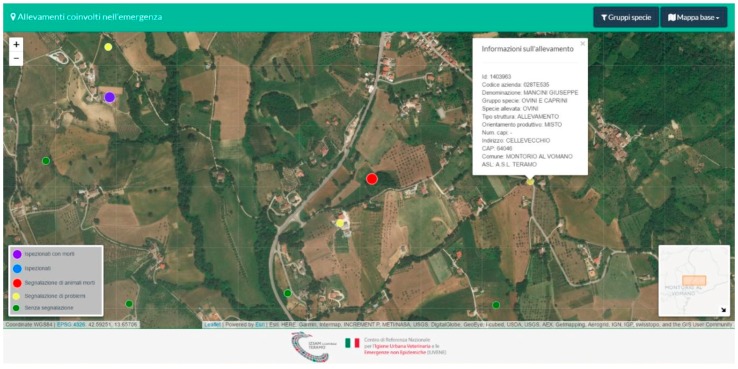
Example of a geolocalized farm for assistance activities. Source: Istituto Zooprofilattico Sperimentale dell’Abruzzo e del Molise “G. Caporale”.(The assistance operations through livestock feed supply were completed on 2 March, 2017.).

IUVENE also supported the military forces by providing a detailed list of interventions to be carried out on a local basis. This information was based on the requests for assistance received and on the position and altitude of the areas to be reached. Maps were developed for helicopters (Figure 4) and farms were geolocalized by converting the decimal coordinates extracted from BDN into sexagesimal data (DMS).

## 4. Discussion

The impact of the same disasters might be very different from region to region and from community to community. During a disaster, people lose their property and these events have negative impacts in poorer and less resilient sections of a community, especially in developing countries, as their dependence on animals for livelihood are invariably greater [9]. Likewise, the problem is even worse when animals are not included in preparedness, mitigation, or rehabilitation plans [9].

In terms of the seismic events that occurred in central Italy in 2016 and 2017, the National Reference Center for veterinary urban hygiene and non-epidemic emergencies—IUVENE—made available all the operational tools to support those managing the emergency. The use and continuous updating of the IUVENE information system, punctual data management, along with the technical support that was provided to the participants operating under the CCS, the LVS and the CUTFAA of Carabinieri, were essential for the rationalization of resources and the organization of the interventions.

**Figure 5:**
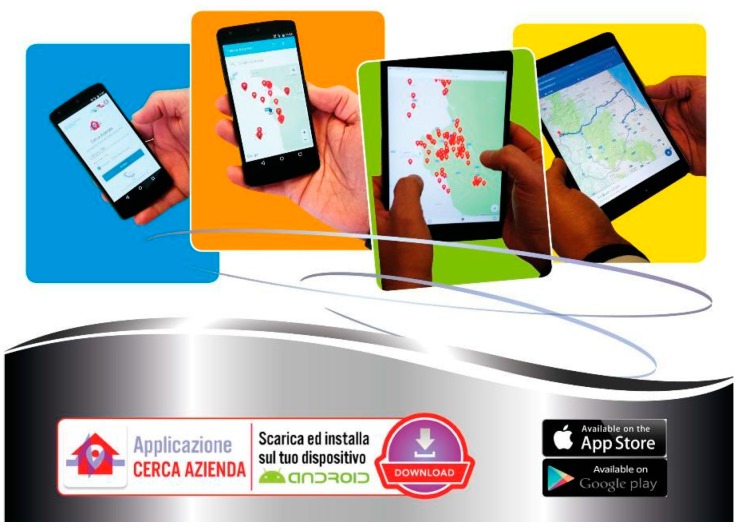
Mobile application called “Search the Farm”, Source: Istituto Zooprofilattico Sperimentale dell’Abruzzo e del Molise “G. Caporale”.

Indeed, assistance and recovery activities for non-epidemic emergencies are often constrained by logistical problems, leading to delays. Often this is due to a lack of readily available data that is needed to assess and define the challenges in real time. Such delays in the application of means and resources to support LVS, which are often directly affected by the disaster, can lead to serious consequences for animal health and welfare as well as for the restoration of normal economic activities for affected agribusinesses. This new, unprecedented challenge to the livelihood of local small scale farmers underlines the need to coordinate an approach to disaster management in rural communities in a holistic, unified manner. On the basis of this experience, IUVENE is proceeding with a further development of the information system, which could be deployed for the collection and analysis of data generated during veterinary activities that are undertaken in any future non-epidemic emergencies. To this end, in addition to the development of the Web-GIS (geographic information system) and the definition of specific information layers, new applications have been developed through mobile devices (smartphones and tablets) to be used by the LVS to operate directly in the field, as well as by other types of users who could take advantage of interactive online services. The mobile application called “Search the Farm”, would allow rescuers (Figure 5) to move on the ground in a quick and effective way and reach farms to be inspected, even without a thorough knowledge of the territory.

To date, the availability of seismic damage scenarios remains of critical importance in order to be able to quickly estimate the expected damage as well as the nature of the resources to be allocated for rescue and emergency management. The future challenge will be the development of a new information system for the assessment of non-epidemic emergency scenarios in veterinary public health. For this purpose, the updating of the emergency management system will require a review of the data available on the real vulnerability of companies, such as those on the type of structure and building materials, in relation to the micro-seismic data of the territory, to identify specific risk factors for farms and livestock. This will, in turn, strengthen the response capacity by improving the risk prevention and mitigation phases. This innovative system represents an additional strategic tool at local, regional, and national levels supporting the development and implementation of the LVS non-epidemic emergency management plans, and as such will be made available to the National Civil Protection system. Through a multidisciplinary approach, multidisciplinary professional skills and competencies, specifically dedicated to non-epidemic emergency management, it will be possible to define empirical and analytical models for assessing damage scenarios stemming from data and information obtained by existing veterinary information systems.

## 5. Conclusions

The events occurred in Abruzzo in January 2017 underscore the importance of the need to coordinate a holistic, cross-agency approach for a successful disaster management in rural communities. Also, in such times of emergency the development of new information systems is important in order to improve the efficiency and effectiveness of disaster-handling activities. Data presented here served to measure the damage and identify those basic needs of the affected population and animals that require immediate response. We are hopeful that decision-making will be aided by the availability of these information.

## Figures and Tables

**Table 1:** Distribution of the farms that reported collapsed stables in the territory of Abruzzo region.

Province	Number of Collapsed Farms
L’Aquila	21
Chieti	2
Pescara	13
Teramo	246
Total	282

**Table 2:** Support functions and operational structures of the National Department of Civil Protection—NCPD.

NDPC—SUPPORT FUNCTIONS	NDPC—OPERATIONAL STRUCTURES
F1 Planning and technique F2 Health, social and veterinary assistance F3 Media and information F4 Volunteers F5 Means and materials F6 Transportation and viability F7 Telecommunications F8 Essential services F9 Damage assessment F10 Operative structures F11 Local authorities F12 Dangerous materials F13 Assistance to the population F14 Coordination of operational centers.	National groups of scientific research National technical services Voluntary service Fire Department Armed Forces Forestry Commission Police Forces Italian Red Cross National Health Service National Corps for Mountain and Speleological Rescue

**Table 3:** Assistance calls received by National Reference Center for Veterinary Urban Hygiene and Non-Epidemic Emergencies (IUVENE) help desk.

Categories of Requests	Total	Done/Resolved	In Progress	To Work Out
Request for livestock feed supply	265	200	15	50
Damaged stable and request for livestock feed supply	85	77	1	7
Report of logistic difficulties	72	54	5	13
Damaged stable	40	32	1	7
Damaged stable and dead farm animals	36	33	1	2
Health intervention or supply of veterinary medicinal products	26	18	0	8
Help offer (livestock feed, veterinary products, various materials and stables)	19	6	7	6
Adoption or hospitality offer for pets and farm animals	18	1	1	16
Damaged stable, stray animals and livestock feed supply	12	11	1	0
Request for carcasses disposal	12	9	1	2
Stable not reachable due to the snow	9	7	1	1
Presence of stray animals	8	1	4	3
Availability to volunteer	5	0	0	5
Damaged stables and blessed farm animals	5	5	0	0
Damaged stables, blessed farm animals and livestock feed supply	4	4	0	0
Lost animal rescue	4	3	0	1
Damaged stable, blackout and dead animals	3	3	0	0
Electric blackout	3	3	0	0
Damaged stable and electric blackout	3	3	0	0
Damaged stable and stray animals	2	2	0	0
Call for information	1	0	1	0
Money donation offers	1	0	1	0
Total calls	633	472	40	121
